# Relationship between HLA-DRB1 gene polymorphism and breast cancer: A protocol for systematic review and meta-analysis

**DOI:** 10.1097/MD.0000000000025078

**Published:** 2021-03-26

**Authors:** Linlin Liu, Xu Sun, Chenxi Yuan, Huaimin Liu

**Affiliations:** Department of Integrated TCM and Western Medicine, Affiliated Cancer Hospital of Zhengzhou University, Zhengzhou, Henan Province, China.

**Keywords:** breast cancer, gene polymorphism, human leucocyte antigen DRB1, protocol, systematic review

## Abstract

**Background:**

Breast cancer is one of the common malignant tumors in women, which seriously affects women's physical and mental health and even life-threatening. The occurrence and development of breast cancer are closely related to genetic factors. Many studies have shown that human leukocyte antigen DRB1 is associated with the development of breast cancer, but lack evidence. This study aims to systematically evaluate the relationship between HLA-DRB1 gene polymorphism and breast cancer.

**Methods:**

The retrieval time of this study was from the establishment of the database to February 2021. The retrieval databases included CNKI, Wanfang, VIP and China Biomedical Database, PubMed, Embase, Web of Science, and the Cochrane Library. The retrieval objects were observational studies on the relationship between HLA-DRB1 gene polymorphism and breast cancer (including case--control studies, cross-sectional studies, and cohort studies). The language restrictions were English and Chinese. Two researchers independently extracted the data and assessed the quality of the included studies, and Stata 16.0 software was used for statistical analysis.

**Results:**

This study will systematically evaluate the relationship between HLA-DRB1 gene polymorphism and breast cancer based on existing studies.

**Conclusion:**

This study will explore the early warning signal of breast cancer genetic susceptibility, and provide evidence-based medical evidence for clarifying the role of HLA-DRB1 gene polymorphism in breast cancer.

**OSF Registration number:**

DOI 10.17605/OSF.IO/847FQ

## 1 Introduction

Breast cancer is one of the common malignant tumors in women. According to the 2012 global cancer report data^[1]^ released by International Agency for Research on Cancer (IARC), breast cancer accounts for the first place in the incidence of female cancer. There are about 1.671 million new cases of breast cancer in the world each year, and about 5.22 million cases die of breast cancer each year, which seriously endangers women's health. The epidemiological characteristics of breast cancer have obvious regional differences, ethnic and ethnic differences, and family history, which are similar to the genetic characteristics of human leukocyte antigen (HLA).^[2]^ About 5% to 10% of breast cancer cases have familial genetic tendency, and the first-degree relatives have a high incidence of breast cancer.^[3]^ Under the same environmental exposure level, people with different genetic backgrounds have different responses to the exposure environment, indicating that different genetic backgrounds may be protective or hazardous factors for the occurrence of breast cancer in individuals. HLA allele polymorphism is an essential genetic factor that determines the ability of immune response, and different HLA allele polymorphism will affect the degree of individual immune response.^[4]^

HLA system is the major histocompatibility complex (MHC), the system is located in human chromosome 6, is the decisive factor of immune response.^[5]^*HLA-DRB1* gene has the most abundant polymorphism in *HLA-II* gene, which is the decisive factor of immune antigen polymorphism. It is mainly involved in the immune process of tumor body and affects the occurrence and development of various tumor diseases.^[6]^

At present, the association between HLA-DRB1 allele polymorphism and breast cancer has attracted many scholars’ attention. Many studies have explored the relationship between HLA-DRB1 allele polymorphism and breast cancer.^[7–10]^ However, as HLA-DRB1 is rich in polymorphism, there are both correlations and differences among people of different races, nationalities, and regions, the conclusions obtained by different studies are not identical or even opposite. Therefore, we adopted the meta-analysis method to comprehensively analyze the existing data in order to provide evidence-based medicine evidence for clarifying the relationship between HLA-DRB1 allele polymorphism and breast cancer genetic susceptibility.

## 2 Methods

### 2.1 Protocol register

This protocol of systematic review and meta-analysis has been drafted under the guidance of the preferred reporting items for systematic reviews and meta-analyses protocols (PRISMA-P).^[11]^ Moreover, it has been registered on open science framework (OSF) (Registration number: DOI 10.17605/OSF.IO/847FQ).

### 2.2 Ethics

As the protocol did not require patient recruitment and the collection of personal information, it did not require approval from an ethics committee.

### 2.3 Eligibility criteria

(1) Studies where the relationship between HLA-DRB1 gene polymorphism and breast cancer was studied and evaluated;
(2) Observational studies (including case--control studies, cross-sectional studies, cohort studies);
(3) Studies with allele or genotype distribution frequency data available;
(4) Studies with the distribution frequency of genotypes conformed to Hardy--Weinberg law.

### 2.4 Exclusion criteria

(1) Republished studies;
(2) Published literatures for summary or review, articles with incomplete data or data errors, and contact the author cannot get complete data of the article;
(3) Failure to provide detailed genotype frequency data;
(4) Literatures without related outcome indicators;
(5) The research object is not from human.

### 2.5 Search strategy

“HLA-DRB1,” “ gene polymorphism,” and “ breast cancer” were used as Chinese search terms to search in Chinese databases, including CNKI, Wanfang Data Knowledge Service Platform, VIP Chinese Journal Service Platform and Chinese Biomedical Database; “HLA antigen,” “HLA-DRB1,” “polymorphism,” “breast cancer,” “breast tumor,” etc, were searched in English databases, including PubMed, EMBASE, Web of Science, the Cochrane Library. All the domestic and foreign literatures on the relationship between HLA-DRB1 gene polymorphism and breast cancer were collected from the establishment of database to February 2021. Taking PubMed as an example, the search strategy is shown in Table 1.

**Table 1:** Search strategy in PubMed database.

Number	Search terms
#1	HLA antigen [Title/Abstract]
#2	HLA-DRB1 [Title/Abstract]
#3	Human leucocyte antigen DRB1 [Title/Abstract]
#4	#1 OR #2 OR #3
#5	polymorphism [Title/Abstract]
#6	variant [Title/Abstract]
#7	#4 OR #5
#8	Breast cancer [MeSH]
#9	Breast Neoplasm [Title/Abstract]
#10	Breast Tumor [Title/Abstract]
#11	Mammary Cancer [Title/Abstract]
#12	Malignant Neoplasm of Breast [Title/Abstract]
#13	Malignant Tumor of Breast [Title/Abstract]
#14	Cancer of Breast [Title/Abstract]
#15	Mammary Carcinoma, Human [Title/Abstract]
#16	Human Mammary Neoplasm [Title/Abstract]
#17	Breast Carcinoma [Title/Abstract]
#18	#7 OR #8 OR #9 OR #10 OR #11
#19	#4 AND #7 AND #18

### 2.6 Data screening and extraction

**Figure 1:**
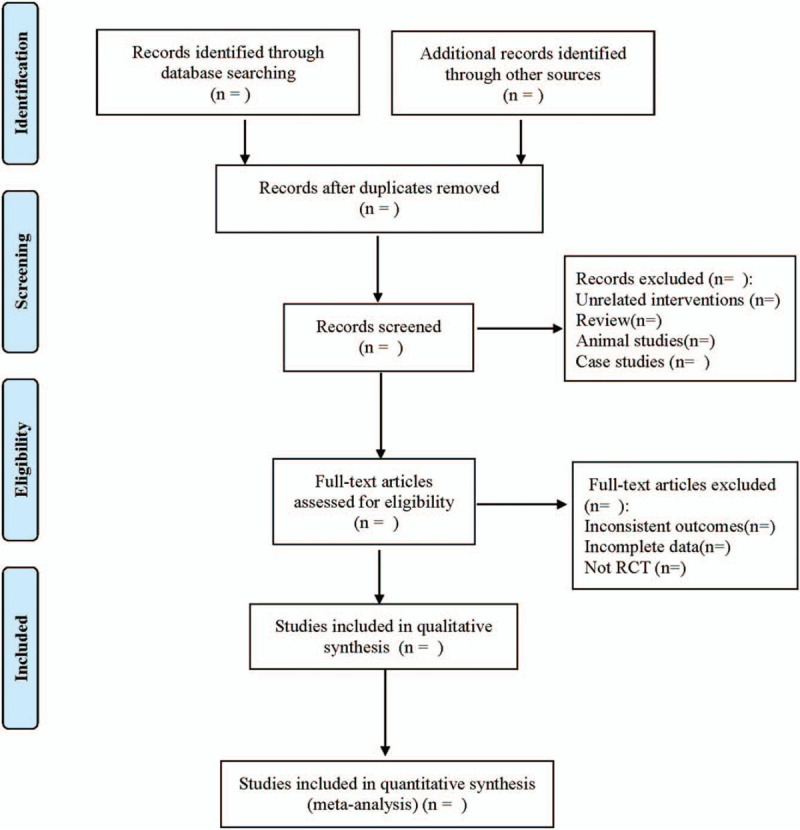
Flow diagram.

Two researchers independently completed literature screening. After excluding the studies that obviously did not meet the inclusion criteria, they further read the abstract and full text to determine whether they met the inclusion criteria. Data from the included literatures were extracted and cross-checked. In case of disagreement, consult with the third researcher and reach a consensus. The extracted data include the first author, publishing year, publishing country, ethnicity of the study population, basic characteristics of the study population (including age, sex, disease, etc), distribution of each gene phenotype (whether it complies with Hardy-Weinberg equilibrium law), and detection method of gene polymorphism. The literature screening process is shown in Figure 1.

### 2.7 Literature quality assessment

The quality of case--control and cohort studies was evaluated by the Newcastle--Ottawa Scale (NOS),^[12]^ including 3 columns and 8 items with a total score of 9, and the evaluation criteria ≥6 was high quality; The cross-sectional studies were evaluated using 11 standard entries from the evaluation cross-sectional studies recommended by the U.S. Agency for Healthcare Research and Quality (AHRQ); 0 ∼ 3 is low quality, 4 ∼ 7 is medium quality, and 8 ∼ 11 is high quality.^[13]^

### 2.8 Statistical analysis

#### 2.8.1 Data analysis and processing

Before evaluating the correlation between gene polymorphism and breast cancer, the genotype distribution of each control group was tested for Hardy--Weinberg genetic balance (*P* ≥ .05). The software STATA.16 was used for meta-analysis, continuity variables were expressed as standard mean difference (SMD) and 95% confidence interval (CI), while binary variables were expressed as odds ratio (OR) and 95% CI. Q test and *I*^*2*^ were used for heterogeneity analysis, and *I*^*2*^ value was used to evaluate the magnitude of heterogeneity. If *P* > .1 and *I*^*2*^ < 50%, it indicated that the heterogeneity among included studies was small, and the fixed effect model was used for analysis. If *P* < .1 and *I*^*2*^ ≥ 50%, it suggested obvious heterogeneity among included studies. The source of heterogeneity was analyzed and the random effect model was used for analysis.

#### 2.8.2 Dealing with missing data

If the data are incomplete or not reported in the study, the researcher will contact the first author or other authors of these studies by telephone or email. If we cannot get the required data, we will use descriptive analysis instead of meta-analysis, if necessary, eliminate these studies.

#### 2.8.3 Subgroup analysis

In order to address the heterogeneity between studies, subgroup analyses were conducted according to race (e.g., yellow, white, etc), region (e.g., European, Asian, etc) and population (high-risk populations with hypertension, diabetes, heart disease, and healthy low-risk populations).

#### 2.8.4 Sensitivity analysis

To test the stability of meta-analysis results, we use Stata 16.0 to perform sensitivity analysis using one-by-one exclusion.

#### 2.8.5 Assessment of reporting biases

We will use funnel plot to qualitatively identify publication bias, and use Egger and Begg test to quantitatively evaluate publication bias. If the funnel plot is asymmetric and *P* < .05, it is considered to have obvious publication bias.

#### 2.8.6 Evidence quality evaluation

For studies that can achieve meta-analysis, we will use the Grading of Recommendation Assessment, Development and Evaluation (GRADE) scoring method to grade the evidence of its outcome indicators.^[14]^ Evaluation includes risk of bias, indirectness, inconsistency, inaccuracy, publication bias, and the quality of evidence will be rated as high, medium, low, or very low.

## 3 Discussion

Breast cancer is the most common cancer among women, and the incidence of breast cancer is increasing globally due to factors that are not fully understood. The pathogenesis of breast cancer is complex, and the relationship between gene polymorphism and the risk of breast cancer has attracted extensive attention. Precision therapy is the direction of breast cancer treatment at present. With the increasing attention of HLA in tumor immunity, we can find suitable tumor specific antigen peptide as tumor vaccine, and provide new methods and ideas for the prevention and treatment of breast cancer.^[15]^

HLA is the most complex and polymorphic gene in human, which is related to the occurrence of many kinds of tumors, and has always been a hot in tumor immunity research.^[16,17]^ HLA consists of closely linked I, II, III genes. HLA-DRB1 is the most polymorphic locus of HLA-II genes, it is mainly involved in the immune process of tumor body,^[18]^ mainly expressed on the surface of APC cells (macrophages, dendritic cells, and B cells). It presents exogenous antigens to CD4^+^ regulatory T cells, helper T cells (Th) and inhibitory T cells (Ts) and participates in the immune response process.^[19]^ HLA-DRB1, as an immune effector molecule, plays an important role in the occurrence and development of breast cancer. By studying the association of HLA-DRB1 and breast cancer, it is helpful for early diagnosis, early treatment, and prognosis of breast cancer.

At present, there have been studies on the correlation between *HLA-DRB1* gene polymorphism and breast cancer. Because of the different genetic background, environmental factors, and research methods, the regional differences are also great. Studies suggest that DRB1∗12, DRB1∗1801, and DRB1∗1501 are highly expressed in breast cancer populations in Iran, Mexico, Istanbul, and Greece, respectively.^[20]^ Studies have also found that DRB1∗01, DRB1∗13, and DRB1∗15 are not associated with risk of breast cancer in Jordan.^[21]^ Ghaderi et al^[22]^ have found that HLA-DRB1∗12 are susceptible genes to breast cancer, while Gun et al^[23]^ found that HLA-DRB1 ∗ 03 is a protective gene of breast cancer. The reason for inconsistency is that the protocol itself is still affected by population characteristics or other factors. This study will explore the relationship between HLA-DRB1 gene polymorphism and breast cancer by systematic review and meta-analysis.

Due to the limitation of language retrieval, we only included Chinese and English literature in this study, and may ignore the research of other languages. Factors such as race, skin color, and disease may cause some clinical heterogeneity.

## Author contributions

**Data collection:** Linlin Liu and Xu Sun

**Data curation:** Linlin Liu, Xu Sun.

**Funding acquisition:** Huaimin Liu.

**Funding support:** Huaimin Liu

**Resources:** Xu Sun and Chenxi Yuan

**Software operating:** Chenxi Yuan and Huaimin Liu

**Software:** Chenxi Yuan, Huaimin Liu.

**Supervision:** Chenxi Yuan.

**Writing – original draft:** Linlin Liu and Xu Sun

**Writing – review & editing:** Linlin Liu and Huaimin Liu
